# Long term outcomes of pituitary adenomas in Multiple Endocrine Neoplasia type 1: a nationwide study

**DOI:** 10.3389/fendo.2024.1427821

**Published:** 2024-10-08

**Authors:** Nuria Valdés, Ana Romero, Estrella Diego, María Calatayud, Cristina Lamas, Marta Araujo-Castro, Cristina Álvarez-Escolá, José A. Díaz, Victoria Alcázar, Julia Sastre, Rosa Martínez, Josep Oriola, Miguel Paja, Paula Sánchez-Sobrino, Isabel Salinas, José María Recio-Córdova, Elena Navarro, María Dolores Chiara, Luis Castaño, Anna Casterás

**Affiliations:** ^1^ Endocrinology & Nutrition Department, Hospital Universitario Cruces, Barakaldo, Bizkaia, Spain; ^2^ Biobizkaia Health Research Institute, Hospital Universitario Cruces, University of the Basque Country, CIBERDEM, CIBERER, Endo-ERN, Barakaldo, Bizkaia, Spain; ^3^ Endocrinology & Nutrition Department, Hospital Universitario Virgen del Rocío, Sevilla, Spain; ^4^ Endocrinology & Nutrition Department, Hospital Universitario Cruces, Barakaldo, Spain; ^5^ Endocrinology & Nutrition Department, Hospital Universitario Doce de Octubre, Madrid, Spain; ^6^ Endocrinology & Nutrition Department, Hospital Universitario de Albacete, Albacete, Spain; ^7^ Endocrinology & Nutrition Department, Hospital Universitario Ramón y Cajal, Instituto de Investigación Biomédica Ramón y Cajal (IRYCIS), Madrid, Spain; ^8^ Endocrinology & Nutrition Department, Hospital Universitario La Paz, Madrid, Spain; ^9^ Endocrinology & Nutrition Department, Hospital Universitario San Carlos, Madrid, Spain; ^10^ Endocrinology & Nutrition Department, Hospital Universitario Severo Ochoa, Madrid, Spain; ^11^ Endocrinology & Nutrition Department, Hospital Universitario de Toledo, Toledo, Spain; ^12^ Endocrinology & Diabetes Research Group, Hospital Universitario Cruces, Barakaldo, Spain; ^13^ Biochemistry and Molecular Genetics Department, Hospital Clínic Universitari, IDIBAPS, Barcelona, Spain; ^14^ Endocrinology & Nutrition Department, Hospital Universitario de Basurto, Bilbao, Spain; ^15^ Endocrinology & Nutrition Department, Complexo Hospitalario Universitario de Pontevedra, Pontevedra, Spain; ^16^ Endocrinology & Nutrition Department, Hospital Universitario Germans Trias i Pujol, Barcelona, Spain; ^17^ Endocrinology & Nutrition Department, Hospital Universitario de Salamanca, Salamanca, Spain; ^18^ Institute of Sanitary Research of the Principality of Asturias (ISPA), Oviedo, Spain; ^19^ Institute of Oncology of the Principality of Asturias, University of Oviedo, Oviedo, Spain; ^20^ Endocrinology & Nutrition Department, Hospital Universitario de Vall d´Hebron, Barcelona, Spain

**Keywords:** pituitary adenomas, Multiple Endocrine Neoplasia type 1, non-functioning pituitary adenomas, prolactinomas, outcomes, microadenomas, macroadenomas, progression

## Abstract

**Introduction:**

Historically, Multiple Endocrine Neoplasia type 1 (MEN1)-related pituitary adenomas (PAs) were considered more aggressive and treatment-resistant than sporadic PAs. However, recent studies suggest similarities in their behavior. This study aimed to evaluate the long-term outcomes of MEN1 PAs and identify predictive factors.

**Methods:**

Nationwide multicenter retrospective cohort study of MEN1-related PAs with a minimum 1-year follow-up, collecting patient demographics, germline *MEN1* pathogenic variants (PV), PA size, secretory profile, radiological characteristics, treatments, and outcomes.

**Results:**

We analyzed 84 PAs, 69%in females and 31% in males (P<0.001), diagnosed at a mean age of 35.2±14.9 years, mostly through screening (60.7%). Median follow-up was 9 years (IQR:4-16). Prolactin-secreting PAs (PRLomas) (53.5%) and microadenomas (65.5%) were most common. Dopamine agonist treatment was first line for 16 macroPRLomas and 25 microPRLomas, 60.9% of them achieved PRL normalization. There was no significant association observed with tumor size, sex, treatment duration or *MEN1* PV. The risk of progression from micro-PA to invasive macro-PA was 7.2% (4/55), after 8 years (IQR:4-13), all of them were microPRLomas. Kaplan-Meier estimation curve showed significantly higher progression probability in microPRLomas than in other microadenomas subtypes (P=0.017) or microNFPAs (P=0.032). No differences were found between sex, age, or germline *MEN1* PV.

**Conclusion:**

MEN1-related micro-PAs have a low risk of progressing to invasive macro-PAs, regardless of sex, age at diagnosis, or *MEN1* germline PV. The risk is higher for microPRLomas over the long term. Therefore, long-term surveillance with reduced frequency, rather than intensive short-term monitoring, may be appropriate for patients with MEN1-related PAs.

## 1 Introduction

Multiple Endocrine Neoplasia type 1 (MEN1) is a genetic condition that is manifested as a constellation of multiple endocrine tumors. The hallmark of this autosomal dominant disorder is the presence of parathyroid, enteropancreatic, pituitary, adrenocortical, thymic and bronchial neuroendocrine tumors. Among the various neoplastic manifestations of MEN1, pituitary adenomas (PAs) are considered a classic diagnostic criterion and one of the three defining lesions of the disease (1, 2). Historically, prolactinomas (PRLomas) were considered the most common type of PAs in MEN1 patients and were thought to have similar behavior and treatment response as PAs that develop in non-MEN1 individuals (3, 4). However, this thinking was revised in light of studies conducted by the French and Belgian MEN1 PAs series (5), the French Group of Endocrine Tumors (GTE) (6), and Burgess et al. (7), which found that MEN1 PAs, particularly PRLomas, were larger, more invasive, and more resistant to treatment compared to sporadic PAs. Conversely, in 2013, a Dutch study of 134 MEN1 PAs (8) found that most PAs diagnosed through screening in MEN1 patients were microadenomas with a natural history similar to that of the general population. This finding was further supported by cohort studies from the Mayo Clinic Group (9) and again by the French GTE (10), suggesting a generally less aggressive nature of MEN1 PAs than previously anticipated.

Recent findings underscore the significant role that the diagnosis of PAs plays as a predictor of life quality in MEN1 patients, making it as the second most consistent determinant (11). Since 2014, concerns have also been raised regarding the potential for gadolinium-based contrast agents, commonly used in magnetic resonance imaging (MRI), to accumulate in brain ganglia, the implications of which remain to be fully understood (12). Given these considerations, it is essential to acquire a comprehensive understanding of the behavioral patterns of MEN1-associated PAs to guide the formulation of optimal surveillance strategies. Such strategies must carefully weigh the potential risks associated with gadolinium exposure against the hazards posed by the delayed diagnosis of significant tumor growth, which could complicate treatment efforts. This study aimed to assess the outcomes of PAs in a substantial cohort of MEN1 patients over an extended follow-up period, with a focus on identifying associated risk factors.

## 2 Materials and methods

### 2.1 Clinical studies

This is a multicenter, nationwide study. The study population data was extracted from the Spanish Online Registry of MEN1 and MEN2, and Pheochromocytomas and Paragangliomas (MENPhePar Registry). It was designed by the Spanish MENPhePar Study Group and endorsed by the Spanish Endocrinology and Nutrition Society. Launched in 2007 to collect data on specific manifestations of MEN1 and MEN2, it was modified in 2012 to include more demographic, clinical, and pathological data about these diseases, as well as sporadic and hereditary pheochromocytomas and paragangliomas (13, 14). In Spain, there are no referral centers for these diseases, but most patients are treated in tertiary university hospitals. The Online Registry is open to all members of SEEN, and the doctor responsible for the patient introduces the data.

The Registry was approved by the Ethical Committee of the Principado de Asturias with the reference number N° 45/16. The data were collected both retrospectively and prospectively. Anonymity of patients was preserved. Therefore, the study met all Spanish legal requirements for the use of medical and personal data for scientific research.

According to the current clinical guidelines for MEN1 (1, 2), the diagnosis of MEN1 was based on the presence of at least two out of the three major lesions characteristic of MEN1 (primary hyperparathyroidism, duodenopancreatic neuroendocrine tumor or PA), including patients both with and without germline pathogenic variants (PV). The diagnosis of MEN1 was also established in members of a family with MEN1 who carry the familial germline PV, independently of the presence of MEN1 lesions. Additionally, some index cases were tested due to the presence of atypical MEN1 lesions as defined by the MEN1 guidelines (2).

Genetic testing for *MEN1* germline PV was performed in 237 (98.7%) of the overall cohort, but not in three patients who died before 1999; these patients had at least two canonical neoplasms and were members of a family with a *MEN1* germline PV. Genotype-phenotype correlations were investigated by comparing patients harboring *missense MEN1* PV with patients with other types of PV. Also, we compared patients in whom no PV of the *MEN1* gene could be identified with patients with germline *MEN1* PV.

**Figure 1:**
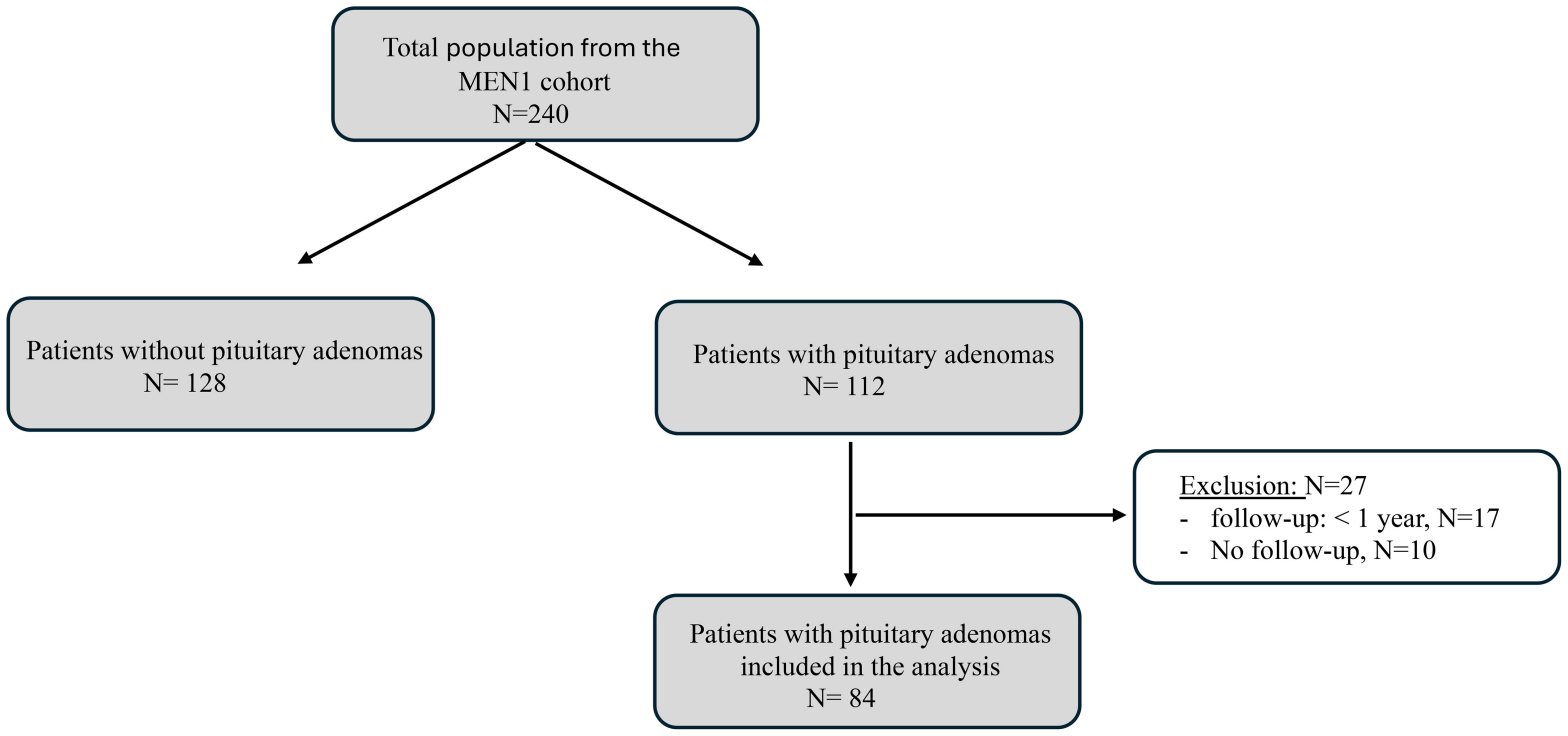
Flowchart of patients selection. MEN1, Multiple Endocrine Neoplasia Type 1.

The inclusion criteria for this study were PAs with or without active treatment with at least one year of follow-up, as it is shown in **Figure 1**. The time period of this study covers from 1980 to 2021. Five patients were diagnosed before 1990, and all of them had MRI during their follow-up. Data were collected on various aspects of MEN1 disease and PAs, including demographic, clinical, radiological, histological, therapeutic, and outcome data. Based on initial imaging, PAs were classified according to Hardy´s classification (HC) (15): grade I: microadenomass (< 10 mm); grade II: enclosed macroadenomas (≥ 10 mm); grades III-IV: localized and diffuse invasion, respectively, both referred to as invasive macroadenomas. PAs were also subclassified into functioning and non-functioning (NFPA) PAs based on laboratory test results.

The evaluation criteria for functioning and NFPAs included an assessment of size increase, specifically the progression from microadenomas or enclosed macroadenomas (HC grades I and II) to invasive macroadenomas (HC grades III and IV) and from microadenomas to invasive macroadenomas. In addition, we also analyzed the PA size reduction. For macroadenomas, this was considered as a reduction in HC, and for microadenomas, any size reduction reported by expert radiologists in the MRI reports. For functioning PAs, control of excessive hormonal secretion with treatment was also considered. Both variables were recorded at the last follow-up. We considered the following prognostic factors for these outcomes: sex, age at PA diagnosis, type of germline *MEN1* PV, and, in the case of PRLomas, the duration of dopamine agonist treatment.

### 2.2 Statistical analysis

Categorical variables were summarized as absolute numbers and percentages. For continuous variables, normality was assessed with the Kolmogorov-Smirnov test. Quantitative normally distributed variables were summarized as mean ± standard deviation (SD) and the range; quantitative non normally distributed variables were expressed as median, interquartile range (IQR). The Student’s t-test was used to compare normally distributed quantitative variables between two groups or categories, while the Mann-Whitney U-test was applied to analyze differences between groups for non-normally distributed quantitative variables. When comparing quantitative variables with more than two groups, the analysis of variance test (ANOVA) was performed with Bonferroni corrections for normally distributed variables, and the Kruskal-Wallis test for non-parametrically distributed variables. Categorical data were compared between groups or categories by Fisher’s exact test. We explored associations between the normalization of PRL levels and reduction of PRLoma size with the following factors: sex, age at PA diagnosis, type of germline MEN1 PV and size (micro or macroadenoma) using logistic regression models, associations were expressed as odds ratios (ORs) with their 95% confidence interval (CI). The Kaplan-Meier survival analysis was used to analyze the progression time to invasive PA and differences between subtypes of PAs, sexes, type of MEN1 germline PV, and different age ranges at PAs diagnosis were tested using the long-rank test. All tests were two-sided, results were considered statistically significant at P<0.05. All statistics were analyzed using the SPSS 28 for Windows (SPSS Inc., Chicago, IL, USA).

## 13 Results

### 3.1 Baseline clinical characteristics

A total of 240 patients with MEN1 were included in the Registry, with 112 (46.6%) diagnosed with PAs. Among those, 84 patients met the inclusion criteria, 58 (69.0%) females and 26 males (31.0%) (P<0.001), with a median follow-up time of 9.0 years (IQR:4-16). MEN1 was diagnosed at 33.1±15.5 years (range:5.0-74), and PAs were detected at a mean age of 35.2±14.9 years (range:13-75). Baseline characteristics are detailed in **Table 1**.

**Table 1:** Characteristics of MEN1-related pituitary adenomas in the entire population and according to secretory profile.

	Entire Population N=84	PRL-secreting adenoma N=45	Non-secreting adenoma N=26	GH-secreting adenoma N=7	ACTH-secreting adenoma N=4	PRL-GH co-secreting adenoma N=2	P
Gender Females (%) Males (%)	58 (69.0) 26 (31)	34 (75.6) 11 (25.0)	17 (65.4) 9 (34.6)	4 (57.1) 3 (42.9)	2 (50) 2 (50)	1 1	0.590
Age at MEN1 diagnosis, years Mean ± SD Range	33.1 ± 15.d5 (5.0-74)	30.4 ± 12.7* (8–72)	33.7 ± 17.8 (5–71)	46.0 ± 16.1 (26–67)	38.5 ± 24.4 (20–74)	22,25	0.299
Age at pituitary diagnosis, years Mean ± SD Range	35.2 ± 14.9 (13–75)	31.8 ± 12.1 (14–67)	37.3 ± 14.1 (13–66)	46.2 ± 16.2 (27–68)	42.0 ± 23.1 (25–75)	23,25	0.814
Index cases (%)	20 (23.8)	15 (33.3)	9 (34.6)	2 (28.6)	3 (75)	1	0.485
Hardy´s classification (%) I: microadenoma II: enclosed macroadenoma III-IV:invasive macroadenoma	55 (65.4) 9 (10.7) 20 (23.8)	26 (57.8) 7 (15.6) 12 (26.7)	23 (88.5) 0 3 (11.5)	2 (28.6) 2 (28.6) 3 (42.9)	3 (75) 0 1 (25)	1 (50) 1 (50)	0.019 0.122 0.340
Type of pathogenic variant (%) None identified Missense Deletion Nonsense Frameshift Splicing	6 (7.1) 12 (14.2) 22 (26.1) 25 (29.7) 13 (15.4) 6 (7.1)	3 (6.6) 5 (11.1) 15 (33.3) 11 (24.4) 7 (15.6) 4 (8.9)	1 (3.8) 4 (15.4) 6 (23.1) 10 (38.4) 3 (11.5) 2 (7.7)	0 2 (28.5) 1 (14.2) 1 (14.2) 3 (42.8) 0	2 (50) 1 (25) 0 1 (25) 0 0	0 0 0 2 0 0	0.022 0.472 0.426 0.117 0.259 0.875
Follow-up, years Median IQR Death during follow-up (%)	9.0 (4–16) 8 (9.5)	9.0 (5.5-18.5) 3 (6%)	6.5 (3.7-13.7) 4 (15.4%)	9.0 (8–13) 0	3 (1.5-24.2) 1 (25.0%)	24, 40 0	0.135 0.475

*Comparison between prolactinomas and GH-producing adenomas, P=0.047.

Thirty-one out of 84 (36.9%) PAs were detected with a median gap of 5.0 years (IQR 2.0-16) before the diagnosis of MEN1, and most of them, 27 (87.1%), were detected in index cases. PAs were diagnosed by screening in 51 (60.7%) MEN1 patients. There was no significant difference in the age at diagnosis between PAs detected before MEN1 diagnosis and those found through screening (39.0 ±16.3 vs 33.0 ±12.1 years, P=0.06).

There were 58 (69.0%) hypersecreting PAs (45 PRLomas, 7 GH secreting adenomas, 4 ACTH secreting adenomas, and 2 PRL-GH co-secreting adenomas) and 26 NFPAs. PRLomas were the most common type of PAs (53.5%), and most of them (60.0%) were diagnosed through screening. Main characteristics according to the secretory profile of PAs and comparative analysis are detailed in **Table 1**.

Concerning PA size, the majority were microadenomass, 55 (65.5%), most of them were microPRLomas (47.3%), and they were more frequently detected through screening (76.4%) (P<0.001). Main characteristics according to PA size and comparative analysis are detailed in **Table 2**.

**Table 2:** Characteristics of MEN1-related pituitary adenomas according to pituitary adenoma size.

	Microadenoma N=55	Macroadenoma N=29	P-value
Gender Females (%) Males (%)	40 (72.7) 15 (27.3)	18 (62.1) 11 (37.9)	0.224
Index cases (%), N=29	15 (27.3)	14 (8.3)	0.047
Age at MEN1 diagnosis, years Mean ± SD Range	30.7 ± 15.3 (5-72)	37.7±15.1 (15-74)	0.048
Age at pituitary diagnosis, years Mean ± SD Range	34.5±13.1 (13-67)	36.5±15.8 (15-75)	0.548
Diagnosis prior to MEN1 diagnosis (%), N=31	13 (41.9)	18 (62.1)	0.001
Diagnosis at first assessment (%), N=33	22 (40.0)	11 (37.9)	0.522
Diagnosis at follow-up (%), N=20	20 (36.4)	0	<0.001
Hormonal secretion (%) Non-secreting pituitary adenoma PRL-secreting pituitary adenoma GH-secreting adenoma ACTH-secreting adenoma PRL-GH co-secreting adenoma	23 (41.8) 26 (47.3) 2 (3.6) 3 (5.5) 1 (1.8)	3 (10.3) 19 (65.5) 5 (17.2) 1 (3.4) 1 (1.8)	0.007 0.103 0.096 0.585 0.230
Type of pathogenic variant (%) None identified Missense Deletion Nonsense Frameshift Splicing	5 (9.0) 6 (10.9) 14 (25.4) 17 (30.9) 9 (16.3) 4 (7.2)	1 (3.4) 6 (20.6) 8 (27.5) 8 (27.5) 4 (13.7) 2 (6.8)	0.244 0.216 0.534 0.457 0.498 0.652
Follow-up time, years Median IQR	8.0 (4.0-1.0)	13.0 (5.5-21.5)	0.067
Death during follow-up (%)	6 (10.9)	3 (10.3)	0.625

### 3.2 Follow-up

**Figure 2:**
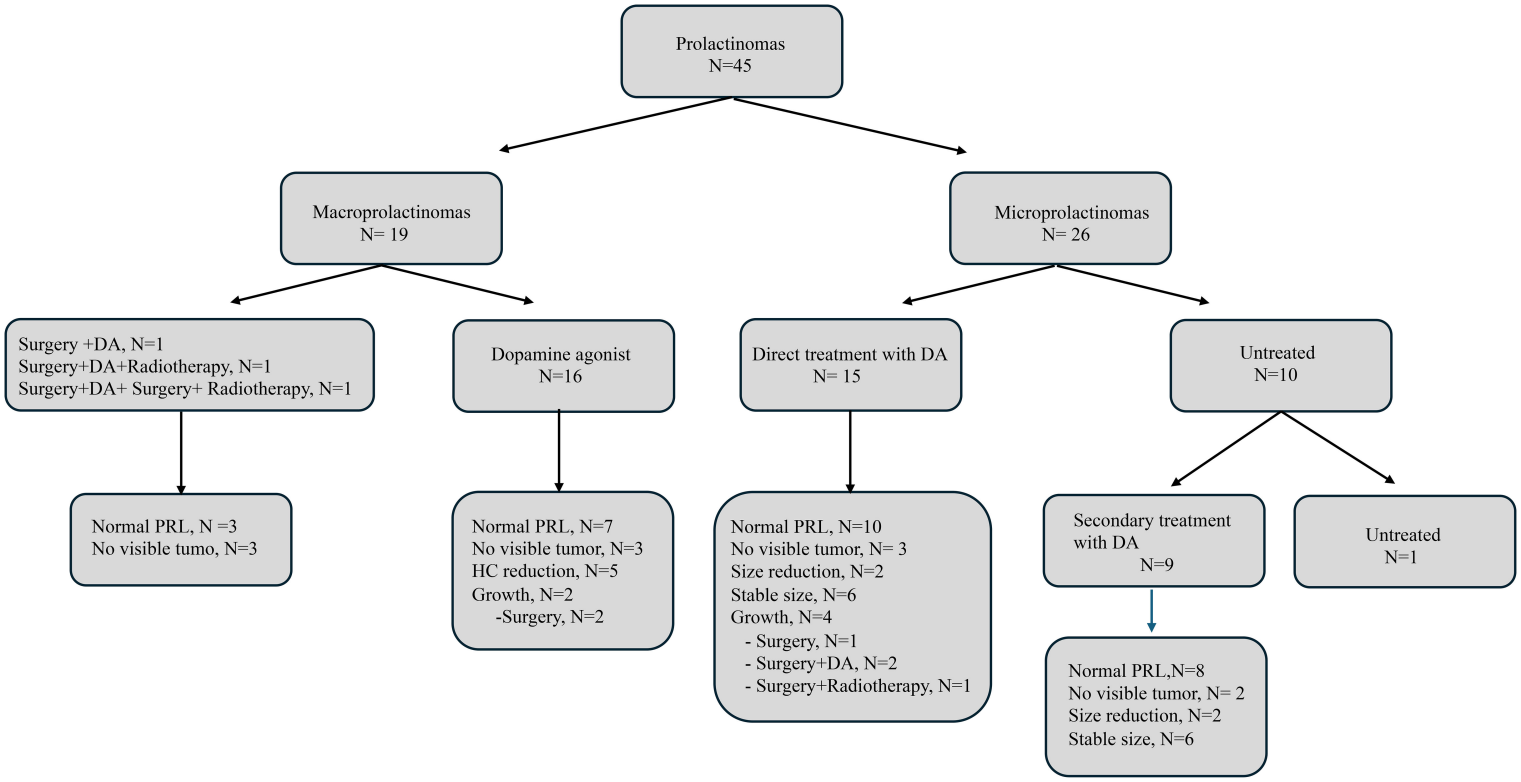
Flowchart of treatment and outcomes for prolactin secreting pituitary adenomas. DA, dopamine agonist; PRL, prolactin; HC, Hardy´s classification.

**Figure 3:**
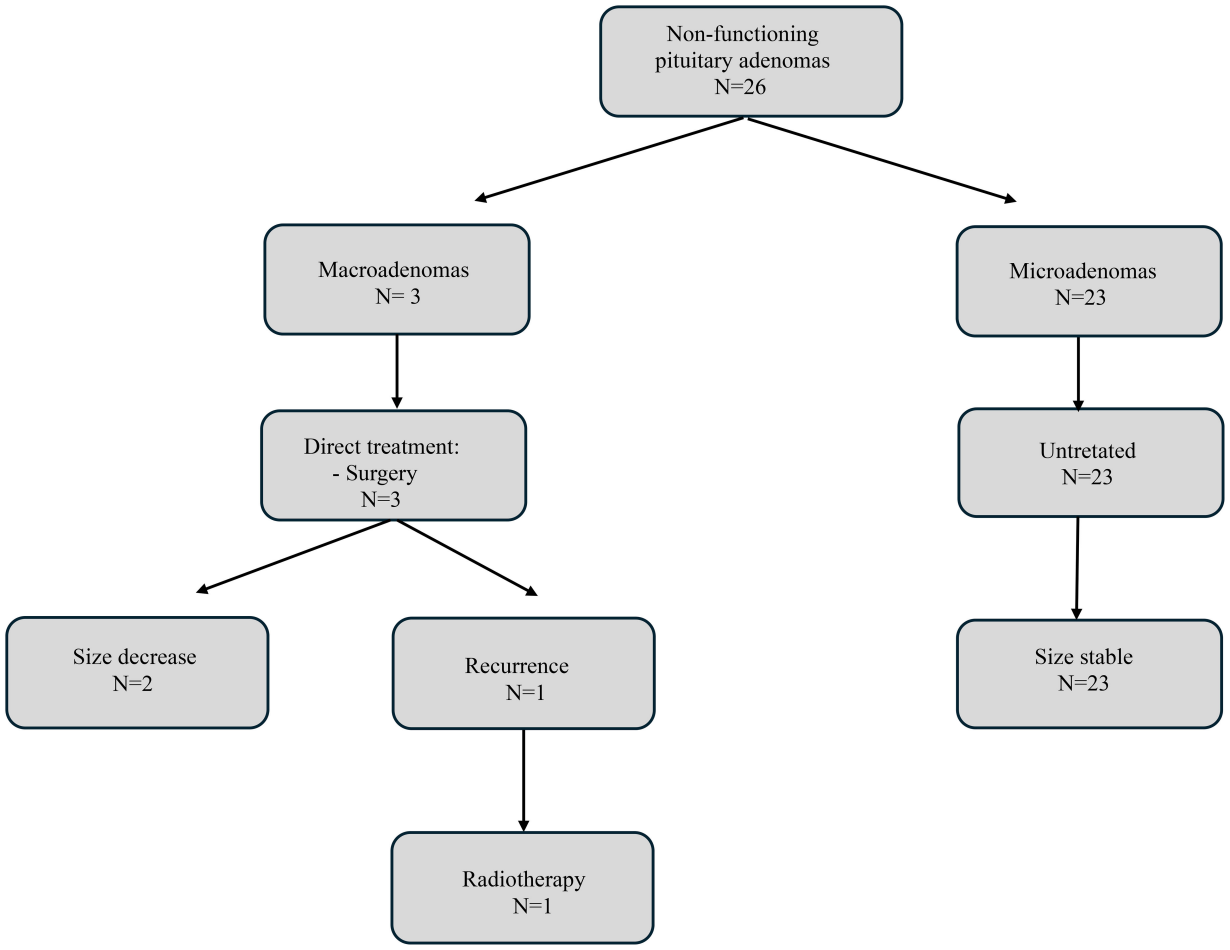
Flowchart of treatment and outcomes for non-functioning pituitary adenomas.

Therapeutic strategies used in our cohort of MEN1-related PAs and their outcomes, according to the type of PA, are shown in **Figures 2**, **3**, and **Supplementary Figures 1**, **2**.

#### 3.2.1 Prolactinomas

##### 3.2.1.1 Macroprolactinomas

There were 19 macroPRLomas, 3 (15.7%) were treated by surgery upon diagnosis, two in 1980 and one in 2002. After surgery, one patient received radiotherapy, and another underwent a second surgery one year later and received radiotherapy due to the size of the remaining tumor. All patients underwent dopamine agonist (DA) therapy following surgery, this being withdrawn at 1,10 and 12 years after surgery, respectively. All these patients displayed normal prolactin (PRL) levels without visible tumor remnants on MRI scan, 18, 34, and 36 years following their diagnosis.

DA treatment was the first-line therapy in the remaining 16 macroPRLomas. After a median follow-up time of 14 years (IQR:8.2-22.2), 7 (43.7%) achieved normalization of PRL levels. Regarding size, 5 (31.2%) showed size reduction as defined by a decrease in HC and 3 (18.7%) patients showed no visible tumor on MRI scan. Despite DA treatment, 2 out of these 16 (12.5%) macroPRlomas increased in their HC (from grade III to IV) and underwent surgery 10 and 17 years after initial diagnosis. No significant differences were observed in size reduction or normalization of PRL levels based on age at PA diagnosis, sex, between enclosed and invasive macroadenomas, duration of medical treatment nor type of *MEN1* germline PV (**Supplementary Tables 1**, **2**).

##### 3.2.1.2 Microprolactinomas

Regarding the 26 micro-PRLomas, DA therapy was started immediately after diagnosis in 15 (57.6%), 10 (38.4%) were treated after a median time of 3.5 years (IQR:16.5), and 1 remained untreated for 8 years. After a median follow-up time of 8.5 years (IQR: 2.7-23.2), 12 out of 25 micro-PRLomas (5%) showed stable size, 5 (20%) showed no visible tumor on the MRI scan, and 4 (16%) showed size reduction. Eighteen (72%) micro-PRLomas achieved normalization of PRL levels. No significant differences were observed in patients’ sex, age at PA diagnosis, treatment duration, treatment initiation timing, or germline *MEN1* PVs when comparing micro-PRLomas that achieved size reduction versus those that did not, and micro-PRLomas that attained normal PRL levels versus those that did not (**Supplementary Tables 2**, **3**). Four cases (15.3%) progressed to invasive macro-PAs after a mean follow-up time of 9.5±2.3 years (range: 8-13), all in females. All were under DA therapy immediately after diagnosis, resulting in normal PRL levels in two patients who were diagnosed at progression with invasive macro-NFPAs. All four patients underwent surgery and one also received radiotherapy. At the last follow-up of 4.5 years (IQR:1-9.5) after invasive macro-PAs diagnosis, all patients showed stable tumor remnant, and the two macro-PRLomas maintained hyperprolactinemia with DA therapy.

Concerning the outcomes of DA therapy as the initial treatment strategy in 41 PRLomas, it was observed that, over an average duration of 10 years, 25 (60.9%) achieved normalized PRL levels, and 17 (41.4%) showed size reduction or became undetectable on MRI. Univariate and multivariate analysis were performed, but neither outcome—normalization of prolactin levels or tumor size reduction—was significantly associated with factors like sex, age, initial tumor size, treatment duration, or type of germline *MEN1* PV (**Supplementary Table 4**).

#### 3.2.2 Non-functioning pituitary adenomas

There were three cases of macro-NFPAs, all of which were invasive. All three patients underwent surgery immediately after diagnosis. One of them had a recurrence of the tumor 3 years after surgery and has just received radiotherapy.

None of the 23 micro-NFPAs showed clinically significant progression during the 8 years (IQR: 4-16) of follow-up while under observation without any treatment.

#### 3.2.3 GH-secreting pituitary adenomas

Three out of seven (42.8%) GH-secreting PA underwent surgery as first-line therapy, all were macro-PAs. Postoperatively, two of them received radiotherapy and one was also treated with medication. Four cases, two macro-PAs and two micro-PAs, received only medical treatment. After a median follow-up time of 9 years (IQR:8-13), 4 (57.1%) macro-PAs showed size reduction according to HC and all patients achieved hormonal control.

#### 3.2.4 ACTH-secreting pituitary adenomas

Among the four patients diagnosed with Cushing disease, three (75%) underwent surgery as first-line therapy, two of whom had micro-PAs. Postoperatively, one patient with a micro-PA required medical therapy and eventually underwent bilateral adrenalectomy. Another patient with a micro-PA refused surgery and was treated with medication. After a median follow-up time of 3 years (range: 1.2-24.2), all patients had cortisol levels in the normal range. Patients who underwent surgery showed no visible tumor on MRI scan, while the patient who was being medically treated had stable PA size.

#### 3.2.5 PRL-GH co-secreting pituitary adenomas

One invasive macro-PA underwent surgery as first-line therapy, postoperatively received radiotherapy, and DA therapy for ten years. After 34 years of follow-up hormone levels were in the normal range and there was no tumoral image on MRI scan. The other PA, which was a micro-PA, was treated with DA therapy, with normalization of the hypersecretion and without change in PA size.

#### 3.2.6 Risk factors for pituitary adenoma progression

We analyzed the risk of PA progression from micro-PA or enclosed macro-PA (HC grades I and II) to invasive macro-PA (HC grades III and IV). Four out of 64 (6.2%) PAs with HC grades I and II had progression after a median follow up time of 8 years (IQR:4-13), all of them were PRLomas (4 out of 33 PRLomas, 12.1%). The Kaplan-Meier estimation curve showed a significantly higher probability of progression in PRLomas compared to other PA subtypes (P=0.039) with no significant differences when PRLomas were compared only with NFPAs (P= 0.070). We did not find differences in the risk of progression between sexes, across different age ranges and germline *MEN1* PV.

The risk of progression from micro-PAs to invasive macro-PAs was 7.2% (4/55), after a median follow-up time of 8 years (IQR:4-13), all of them were micro-PRLomas. As per the Kaplan-Meier estimation curve, micro-PRLomas exhibited a significantly higher probability of progression compared to other subtypes of micro-PAs (P=0.017). Moreover, the cumulative probability of progression in micro-PRLomas was notably higher than in exclusively micro-NFPAs (P=0.032). No significant differences were observed between sexes, across different age ranges and germline *MEN1* PV.

## 4 Discussion

In this nationwide multicenter retrospective study, we aimed to evaluate the natural history and treatment response in 84 MEN1-related PAs. Our study found that the risk of micro-PAs progressing to invasive macro-PAs was low. However, it occurred over a long-term follow-up period and was observed only in micro-PRLomas. These results suggest that a long-term surveillance strategy with reduced frequency, rather than intensive short-term monitoring, may be appropriate for patients with MEN1-related PAs, particularly for those with micro-PRLomas.

Previous studies have employed various criteria to analyze the progression of MEN1-related PAs. Notably, the Dutch series (8) investigated the progression risk of micro-NFPAs with clinical consequences, finding no progression in any of the 39 micro-NFPAs after a median follow-up of 6 years. Similarly, a Chinese study (16) assessed the risk of progression from micro-NFPA to macro-NFPA in 19 cases, none of which converted to macro-PAs after 35 months of follow-up. In contrast, Cohen et al. (9) reported that 10% of 52 NFPAs required surgical intervention after a median follow-up of 8 years, without specifying whether these were micro or macro-NFPAs. Our findings for 23 micro-NFPAs, observed over a longer period (10 years), align with the outcomes reported in the Dutch and Chinese series (8, 16), indicating no progression to macro-NFPA.

Furthermore, the French cohort study (10) specifically examined the progression risk from micro-PA or enclosed macro-PA to invasive macro-PA, including all types of PA, over 3 years, reporting a progression risk of 2.9%. In contrast, our study reveals a higher progression risk of 6.2%, with PRLomas specifically showing an increased risk of 12.1%, compared to 4.1% reported in the French cohort (10). This discrepancy might be attributed to our study’s longer median follow-up period of 9 years, emphasizing that MEN1 PAs may become invasive predominantly after 8 years, as has been found in both studies. Our analysis indicates that micro-PRLomas are significantly more likely to progress to invasive macro-PAs than micro-NFPAs, aligning with findings from the French study (10). Nonetheless, the necessity for distinct monitoring approaches for these two entities can only be conclusively established through a dedicated, prospective, and standardized study.

Taken together, our results and previous studies (8–10, 16), suggest a need to reassess the intensity of MRI surveillance following PA diagnosis in MEN1 cases, because of their characteristically slow growth. This slow growth underscores the need for prolonged follow-up to accurately determine the risk of these PAs becoming invasive. This is in line with recent recommendations for sporadic micro-NFPAs, which advise postponing the initial MRI follow-up to three years post-diagnosis (17–19) and continuing follow-up of these patients over time with reduced frequency (19, 20). Regarding microPRLomas, the recent Pituitary Society international Consensus Statement (21) do not recommend serial imaging beyond 1 year for treatment-responsive cases unless serum levels of PRL persistently increase. However, our findings advocate for ongoing imaging, as a few microPRLomas with normal PRL levels eventually evolved into invasive macroadenomas after a long follow-up. Furthermore, although uncommon, new PAs could occur in MEN1, so surveillance is also important for screening new PAs.

In 1996, Burgess et al. (7) first proposed that PRLomas in MEN1 patients might be more aggressive than their sporadic counterparts. This observation was later supported by a French-Belgian multicenter study on MEN1-related PAs (5), which also noted a diminished effectiveness of DA therapy in these MEN1 PRLomas compared to sporadic cases. Contrasting this, a 2013 Dutch cohort study (8) revealed that DA therapy effectively normalized PRLomas levels in 35 out of 39 (89%) of PRLomas, with 60% being micro-PRLomas, challenging earlier views on their aggressiveness and treatment resistance. Further investigation by Salenave et al. (21), in a study of 77 macro-PRLomas in patients under 20, identified the *MEN1* pathogenic variant as an independent factor predicting DA resistance. Our research, in line with the proportion of micro-PRLomas in the entire cohort of the Dutch study (8), revealed a lower PRL normalization rate compared to their findings, standing at 60.9%.

Although a control group is not available for comparison, the literature provides data on the response to DA therapy of sporadic PRLomas, which vary between 72% to 94% for macro-PRLomas and 78% to 95% for micro-PRLomas according to different DA therapies (22–25). Our results showed lower rates of normalized PRL, 43.75% for macro-PRLomas and 72% for micro-PRLomas, compared to the response rates reported in sporadic PRLomas. Additionally, while sporadic macro-PRLomas typically exhibit a lesser response to DA therapy (23–25), our analysis did not reveal any significant association between tumor size and the rate of PRL normalization.

In our cohort, a higher prevalence of PRLomas was observed in females, which aligns with previous findings reported in both MEN1 (5, 29) and sporadic cases (30). However, we found no discernible differences in PRLoma size or treatment response between males and females. Interestingly, gender-specific variations in PRLoma characteristics have been documented in sporadic cases (26), where males often present with larger, more resistant tumors to DA therapy (27, 28, 31).

Collectively, these findings suggest that MEN1-associated PRLomas, especially macro-PRLomas, might exhibit decreased responsiveness to DA therapy. This underscores the importance of comprehensive studies that include MEN1 patients and control groups with both micro- and macro-PRLomas to definitively understand the efficacy of DA therapy in these cases.

It is important to recognize the limitations of our study, including the most critical limitation, its retrospective nature. As such, it has the drawbacks associated with this type of research, such as potential bias and lack of control over certain variables. Additionally, the data were based on the assessments of referring physicians, which may not be as reliable as data collected by a central core center. Furthermore, the primary outcome was rarely encountered. Moreover, as previous studies in MEN1-related PAs (7–10), we did not have a cohort of control patients with PAs without MEN1. Despite its limitations, our study’s notable strengths include a significantly large cohort of MEN1-related PAs and one of the longest follow-up periods available. This extensive duration is crucial due to the slow growth rate of these tumors. Additional strengths of our study include the involvement of multiple centers and adherence to international clinical guidelines for patient management. These aspects enhance the reliability and generalizability of our findings, offering valuable insights into the effective management of patients with MEN1-related PAs.

## 5 Summary and conclusion

In conclusion, our study involving 84 MEN1-related PAs with extensive follow-up revealed that microadenomas associated with MEN1 generally have a low risk of progressing to macroadenomas, with micro-PRLomas exhibiting a higher risk over a long time. Moreover, we found no significant association with sex, age, or type of *MEN1* germline PV. These findings suggest that a surveillance approach spanning a longer term but with reduced frequency may be more suitable for patients with MEN1-related PAs, especially those with micro-PRLomas. These results contribute to our understanding of the long-term behavior of MEN1-related PAs and underscore the importance of further research, ideally through expansive, multicenter cohorts of MEN1 patients with a long follow-up, to validate and expand upon these insights.

## Data Availability

The raw data supporting the conclusions of this article will be made available by the authors, without undue reservation.

## Supplementary material

Supplementary Figure 1 Flowchart of treatment and outcomes for GH secreting pituitary adenomas. GH, growth hormone; HC, Hardy's classification.

Supplementary Figure 2 Flowchart of treatment and outcomes for ACTH secreting pituitary adenomas. ACTH, adrenocorticotropic hormone.
