# Supplementary figures and images for: Long term outcomes of pituitary adenomas in Multiple Endocrine Neoplasia type 1: a nationwide study
Source: Front Endocrinol (Lausanne). 2024 Oct 8;15:1427821. doi: 10.3389/fendo.2024.1427821 (PMC11493648; doi:10.3389/fendo.2024.1427821)

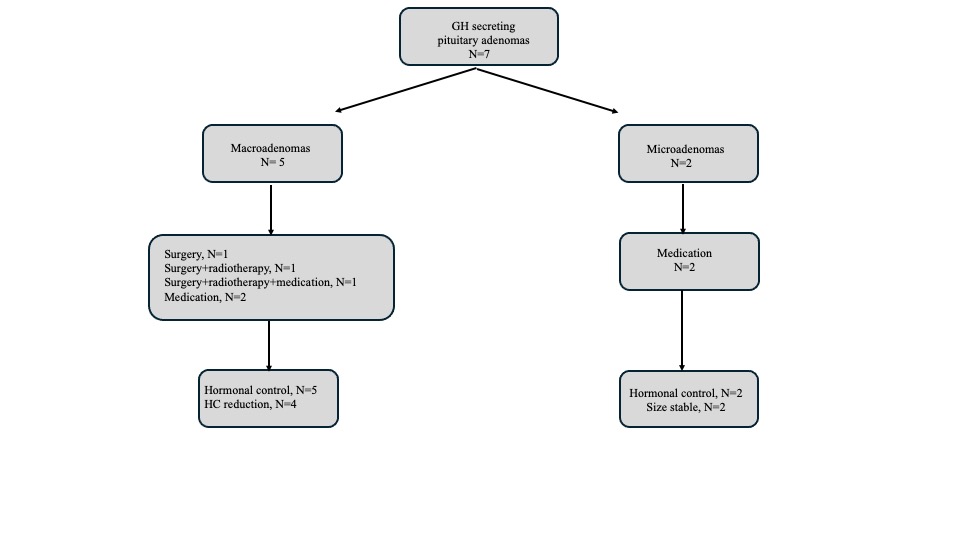

Supplement: Supplementary Figure 1 — Flowchart of treatment and outcomes for GH secreting pituitary adenomas. GH, growth hormone; HC, Hardy's classification. [file Image1.jpeg]

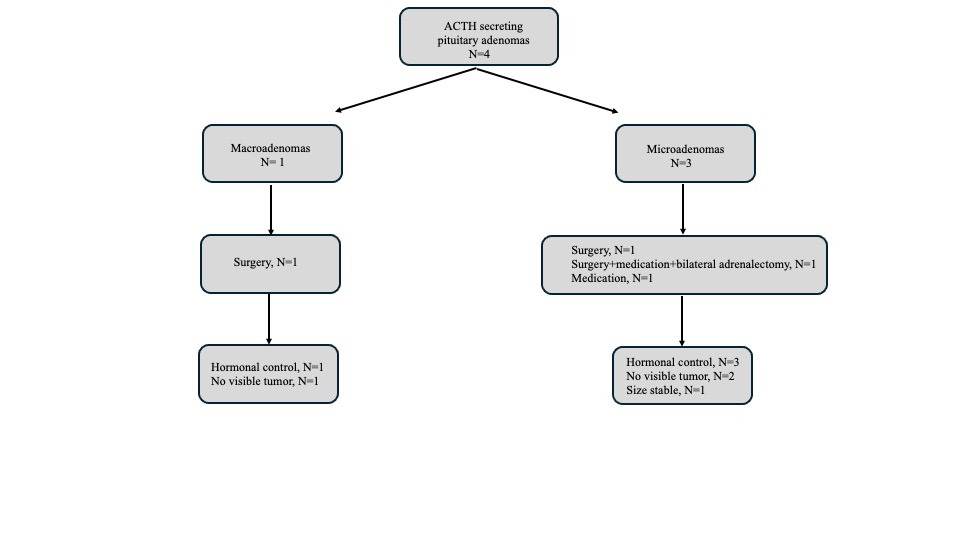

Supplement: Supplementary Figure 2 — Flowchart of treatment and outcomes for ACTH secreting pituitary adenomas. ACTH, adrenocorticotropic hormone. [file Image2.jpeg]
