# Supplementary material for: Long term outcomes of pituitary adenomas in Multiple Endocrine Neoplasia type 1: a nationwide study
Source: Front Endocrinol (Lausanne). 2024 Oct 8;15:1427821. doi: 10.3389/fendo.2024.1427821 (PMC11493648; doi:10.3389/fendo.2024.1427821)
Supplement: Supplementary file 3 [file Table1.docx]

Supplemental Table1. Analysis of variables associated with the normalization of prolactin levels in 16 macroprolactinomas treated with dopamine agonists

|  | Normal prolactin levels  N=7 | Hyperprolactinemia  N=9 | P-Value |
| --- | --- | --- | --- |
| Sex:  Females (%)  Males (%) | 1 (14.3)  6 (85.7) | 2 (22.2)  7 (77.8) | 0.69 |
| Age at pituitary adenoma diagnosis,  years | 28.2 ± 15.0 | 32.4 ± 7.5 | 0.52 |
| *MEN1* germline pathogenic variant:   - *Missense (%)* - *Nonmissense (%)* | 2 (28.6)  5 (71.4) | 1 (11.1)  8 (88.9) | 0.50 |
| Hardy´s classification:   - II (%) - III/IV (%) | 3 (42.8)  4 (57.1) | 3 (33.3)  6 (66.7) | 0.54 |
| Duration of treatment, years | 17.7 ± 10.6 | 12.4 ± 8.0 | 0.27 |

Abbreviations: MEN1: Multiple Endocrine Neoplasia type 1
