# Supplementary material for: Long term outcomes of pituitary adenomas in Multiple Endocrine Neoplasia type 1: a nationwide study
Source: Front Endocrinol (Lausanne). 2024 Oct 8;15:1427821. doi: 10.3389/fendo.2024.1427821 (PMC11493648; doi:10.3389/fendo.2024.1427821)
Supplement: Supplementary file 4 [file Table2.docx]

Supplemental Table 2. Analysis of variables associated with the reduction of pituitary adenoma size in 16 macroprolactinomas treated with dopamine agonists

|  | Reduction of size  N=8 | No reduction of size  N=8 | P-Value |
| --- | --- | --- | --- |
| Sex:  Females (%)  Males (%) | 6 (75.0)  2 (25.0) | 7 (87.5)  1 (12.5) | 0.50 |
| Age at pituitary adenoma diagnosis, years | 28.5 ± 14.0 | 32.5 ± 8.0 | 0.53 |
| *MEN1* germline pathogenic variant:   - *Missense (%)* - *Nonmissense (%)* | 2 (25.0)  6 (75.0) | 1 (12.5)  7 (87.5) | 0.50 |
| Hardy´s classification:   - II (%) - III/IV (%) | 3 (37.5)  5 (62.5) | 3 (37.5)  5 (62.5) | 0.69 |
| Duration of treatment, years | 16.75 ± 10.2 | 12.7 ± 8.5 | 0.41 |

Abbreviations: MEN1: Multiple Endocrine Neoplasia type 1
