# Supplementary material for: Long term outcomes of pituitary adenomas in Multiple Endocrine Neoplasia type 1: a nationwide study
Source: Front Endocrinol (Lausanne). 2024 Oct 8;15:1427821. doi: 10.3389/fendo.2024.1427821 (PMC11493648; doi:10.3389/fendo.2024.1427821)
Supplement: Supplementary file 5 [file Table3.docx]

Supplemental Table 3. Analysis of variables associated with the normalization of prolactin levels in 25 microprolactinomas treated with dopamine agonists

|  | Normal prolactin levels  N=18 | Hyperprolactinemia  N=7 | P-Value |
| --- | --- | --- | --- |
| Sex:  Females (%)  Males (%) | 13 (72.2)  5 (27.8) | 7 (100)  0 | 0.16 |
| Age at pituitary adenoma diagnosis, years | 33.5 ± 13.1 | 31.4 ± 14.7 | 0.73 |
| *MEN1* germline pathogenic variant  *Missense (%)*  *Nonmissense (%)*  N=22 | 2 (13.3)  13 (86.7) | 0  7 (100) | 0.45 |
| Duration of treatment, years | 8.7 ± 6.1 | 11.2 ± 4.4 | 0.47 |
| Direct treatment:  Yes (%)  No (%) | 10 (55.5)  8 (44.4) | 5 (71.4)  2 (28.5) | 0.39 |

Abbreviations: MEN1: Multiple Endocrine Neoplasia type 1
