# Supplementary material for: Long term outcomes of pituitary adenomas in Multiple Endocrine Neoplasia type 1: a nationwide study
Source: Front Endocrinol (Lausanne). 2024 Oct 8;15:1427821. doi: 10.3389/fendo.2024.1427821 (PMC11493648; doi:10.3389/fendo.2024.1427821)
Supplement: Supplementary file 6 [file Table4.docx]

Supplemental Table 4. Analysis of variables associated with the reduction of pituitary adenoma size in 25 microprolactinomas treated with dopamine agonists

|  | Reduction of size  N=9 | No reduction of size  N=16 | P-Value |
| --- | --- | --- | --- |
| Sex:  Females  Males | 6 (66.7)  3 (33.3) | 14 (87.5)  2 (12.5) | 0.23 |
| Age at pituitary adenoma diagnosis,  years | 37.0 ± 14.1 | 30.6 ± 12.7 | 0.26 |
| *MEN1* germline pathogenic variant:  *Missense (%)*  *Nonmissense (%)*  N=22 | 1 (12.5)  7 (87.5) | 1 (7.1)  13 (92.9) | 0.60 |
| Duration of treatment, years | 9.1 ± 7.0 | 9.6 ± 8.5 | 0.87 |
| Direct treatment:  Yes (%)  No (%) | 5 (55.5)  4 (44.4) | 10 (62.5)  6 (37.5) | 0.53 |

Abbreviations: MEN1: Multiple Endocrine Neoplasia type 1
