# Supplementary material for: Long term outcomes of pituitary adenomas in Multiple Endocrine Neoplasia type 1: a nationwide study
Source: Front Endocrinol (Lausanne). 2024 Oct 8;15:1427821. doi: 10.3389/fendo.2024.1427821 (PMC11493648; doi:10.3389/fendo.2024.1427821)
Supplement: Supplementary file 7 [file Table5.docx]

Supplemental Table 5. Variables associated with normalization of prolactin levels with dopamine agonist treatment in 41 prolactinomas

|  | Univariate | | Multivariate | |
| --- | --- | --- | --- | --- |
|  | Odds Ratio  (95%CI) | P-Value | Odds Ratio  (95%CI) | P-Value |
| Sex:  Males  Females | 1.00  0.45 (0.06-2.31) | 0.372 | 0.48 (0.06-2.55) | 0.448 |
| Age at pituitary adenoma diagnosis, years | 1.00 (0.95-1.05) | 0.992 | 1.00 (0.95-1.05) | 0.992 |
| *MEN1* germline pathogenic variant:  *Nonmissense*  *Missense* | 1.00  3.33 (0.434-69.00) | 0.304 | 2.97 (0.36-87.6) | 0.374 |
| Size:  Macroadenoma  Microadenoma | 1.00  3.30 (0.90-12.98) | 0.075 | 3.18 (0.85-12.8) | 0.139 |
| Duration of treatment | 0.99 (0.92-1.06) | 0.802 | 0.99 (0.92-1.06) | 0.851 |

Abbreviations: MEN1: Multiple Endocrine Neoplasia type 1
