# Supplementary material for: Long term outcomes of pituitary adenomas in Multiple Endocrine Neoplasia type 1: a nationwide study
Source: Front Endocrinol (Lausanne). 2024 Oct 8;15:1427821. doi: 10.3389/fendo.2024.1427821 (PMC11493648; doi:10.3389/fendo.2024.1427821)
Supplement: Supplementary file 8 [file Table6.docx]

Supplemental Table 6. Variables associated with reduction of pituitary adenoma size with dopamine agonist treatment in 41 prolactinomas

|  | Univariate | | Multivariate | |
| --- | --- | --- | --- | --- |
|  | Odds Ratio (95%CI) | P-Value | Odds ratio (95%CI) | P-Value |
| Sex:  Males  Females | 1.00  0.34 (0.06-1.64) | 0.189 | 0.36 (0.06-1.79) | 0.241 |
| Age at pituitary adenoma diagnosis, years | 1.01 (0.96-1.06) | 0.633 | 1.01 (0.96-1.05) | 0.657 |
| *MEN1* germline pathogenic variant:  *Nonmissense*  *Missense* | 1.00  2.30 (0.338-19.40) | 0.393 | 2.21 (0.3021.20) | 0.435 |
| Size:  Macroadenoma  Microadenoma | 1.00  0.563 (0.15-2.014) | 0.377 | 0.57 (0.15-2.10) | 0.574 |
| Duration of treatment | 1.027 (0.95-1.10) | 0.461 | 1.03 (0.96-1.10) | 0.499 |

Abbreviations: MEN1: Multiple Endocrine Neoplasia type 1
